# Genome Analysis of a New *Rhodothermaceae* Strain Isolated from a Hot Spring

**DOI:** 10.3389/fmicb.2016.01109

**Published:** 2016-07-14

**Authors:** Kian Mau Goh, Kok-Gan Chan, Soon Wee Lim, Kok Jun Liew, Chia Sing Chan, Mohd Shahir Shamsir, Robson Ee, Tan-Guan-Sheng Adrian

**Affiliations:** ^1^Faculty of Biosciences and Medical Engineering, Universiti Teknologi MalaysiaSkudai, Malaysia; ^2^Division of Genetics and Molecular Biology, Institute of Biological Sciences, Faculty of Science, University of MalayaKuala Lumpur, Malaysia

**Keywords:** *Rhodothermaceae*, *Rhodothermus*, *Salinibacter*, *Salisaeta*, strain RA, *Rubricoccus*, *Rubrivirga*, *Longimonas*

## Abstract

A bacterial strain, designated RA, was isolated from water sample of a hot spring on Langkawi Island of Malaysia using marine agar. Strain RA is an aerophilic and thermophilic microorganism that grows optimally at 50–60°C and is capable of growing in marine broth containing 1–10% (w/v) NaCl. 16S rRNA gene sequence analysis demonstrated that this strain is most closely related (<90% sequence identity) to *Rhodothermaceae*, which currently comprises of six genera: *Rhodothermus* (two species), *Salinibacter* (three species), *Salisaeta* (one species), *Rubricoccus* (one species), *Rubrivirga* (one species), and *Longimonas* (one species). Notably, analysis of average nucleotide identity (ANI) values indicated that strain RA may represent the first member of a novel genus of *Rhodothermaceae*. The draft genome of strain RA is 4,616,094 bp with 3630 protein-coding gene sequences. Its GC content is 68.3%, which is higher than that of most other genomes of *Rhodothermaceae*. Strain RA has genes for sulfate permease and arylsulfatase to withstand the high sulfur and sulfate contents of the hot spring. Putative genes encoding proteins involved in adaptation to osmotic stress were identified which encode proteins namely Na^+^/H^+^ antiporters, a sodium/solute symporter, a sodium/glutamate symporter, trehalose synthase, malto-oligosyltrehalose synthase, choline-sulfatase, potassium uptake proteins (TrkA and TrkH), osmotically inducible protein C, and the K^+^ channel histidine kinase KdpD. Furthermore, genome description of strain RA and comparative genome studies in relation to other related genera provide an overview of the uniqueness of this bacterium.

## Introduction

The family *Rhodothermaceae* is placed under the order *Cytophagales* of the class *Cytophagia* within the phylum *Bacteroidetes*. At the time of writing, *Rhodothermaceae* consisted of six genera: *Rhodothermus* (Alfredsson et al., 1988; Marteinsson et al., 2010), *Salinibacter* (Antón et al., 2002; Makhdoumi-Kakhki et al., 2012), *Salisaeta* (Vaisman and Oren, 2009), *Rubricoccus* (Park et al., 2011), *Rubrivirga* (Park et al., 2013), and the recently described genus *Longimonas* (Xia et al., 2015). *Rhodothermaceae* is a family of gram-negative, non-sporulating, chemoorganotrophic aerobes shaped as rods or cocci, and most strains are pigmented (Park et al., 2014).

*Rhodothermus marinus* was isolated from a submarine hot spring, at a depth of 2–3 m from the sea surface during low tide (Alfredsson et al., 1988), while *Rhodothermus profundi* was obtained from a deep-sea (2634 m depth) hydrothermal field (Marteinsson et al., 2010). *Rhodothermus obamensis* was initially described as a type strain of *Rhodothermus* (Sako et al., 1996) but was later regarded as a synonym of *R. marinus* owing to high 16S rRNA gene sequence similarity, high DNA–DNA reassociation values, and similar fatty acid profiles (Silva et al., 2000). For *R. marinus* DSM 4252^T^ (Nolan et al., 2009) complete genome is available in GenBank (CP001807). The genome of the sister strain *R. marinus* SG0.5JP17-172 (CP003029) is also available but has not been published. Draft genomes have been deposited for the other strains of *Rhodothermus*, which include *R. profundi* DSM 22212^T^ (BioProject ID: PRJNA303571), *R. marinus* JCM 9785^T^ (PRJDB841), and *R. marinus* SG0.5JP17-171 (PRJNA52953).

Members of the genus *Salinibacter* (*Salinibacter iranicus, Salinibacter luteus*, and *Salinibacter ruber*) were isolated from a hypersaline evaporating water body (Park et al., 2014). *S. ruber* requires a high salt concentration, exhibiting optimum growth in media with 15–25% (w/v) total salts (Antón et al., 2002). In addition, sodium chloride and magnesium chloride are vital for the growth of *S. ruber* (Antón et al., 2002). The complete genome of *S. ruber* DSM 13855^T^ (CP000159.1) has been assembled into one contig of 3,386,737 bp, while the genome of *S. ruber* M8 (PRJNA48827) is available in draft form. A species of another genus, the long, rod-shaped bacterium *Salisaeta longa* S4-4^T^ (= DSM 21114^T^; ATTH00000000.1), was isolated from hypersaline water bodies formed from premixed Dead Sea and Red Sea water samples (Vaisman and Oren, 2009). Although few studies have been conducted on strain S4-4^T^, its genome has been sequenced under the Community Science Program of the Joint Genome Institute (Project ID: 404303).

*Rubricoccus marinus* SG-29^T^, a reddish, coccal bacterium isolated from shallow water of the North Pacific Ocean, is the type strain of the type species of *Rubricoccus* (Park et al., 2011). The average GC content of strain SG-29^T^ (68.9 mol%, determined by HPLC) is the highest value known for *Rhodothermaceae*. Two years after the report of *Rubricoccus marinus*, the same group of researchers isolated another bacterium from deep seawater of the western North Pacific Ocean. They proposed a new genus, *Rubrivirga*, with *Rubrivirga marina* SAORIC-28^T^ as the sole species (Park et al., 2013). Recently, a new genus of *Rhodothermaceae, Longimonas*, was proposed (Xia et al., 2015). *Longimonas halophila* SYD6^T^ was obtained from a marine solar saltern on the coast of Weihai, China (Xia et al., 2015). The 16S rRNA gene sequence similarity between *L. halophila* SYD6^T^ and *S. longa* is less than 92% (Xia et al., 2015). Like most other strains of *Rhodothermaceae*, strain SYD6^T^ is red. At the time of writing, genome information for *Rubricoccus, Rubrivirga*, and *Longimonas* were not available.

Among the six aforementioned genera, *Rhodothermus* and *Salinibacter* are better studied (Mongodin et al., 2005; Peña et al., 2005, 2010; Rosselló-Mora et al., 2008; Oren, 2013; Oren et al., 2016). Because most *Rhodothermus* spp. can grow optimally at 65°C, numerous attempts to mine and examine thermostable proteins from this genus have been reported. Examples of these enzymes include amylase and pullulanase (Gomes et al., 2003), α-l-arabinofuranosidase (Gomes et al., 2000), cellulase (Okano et al., 2014), cellobiose 2-epimerase (Ojima et al., 2011), chitinase (Hobel et al., 2005), and α-galactosidase (Blücher et al., 2000).

In this work, a bacterium designated as strain RA was isolated from a saline hot spring sample. Analyses of the genome, 16S rRNA gene, and housekeeping gene sequences suggested that the strain could represent a new genus of *Rhodothermaceae*. The purpose of this report is to describe the phenotypic characteristics of this strain and present its genome sequence.

## Materials and methods

### Water sample analyses and bacterial characterization

Three water samples were collected from a hot spring near the village of Ayer Hangat (6°25′22″N, 99°48′49″E) and analyzed by Allied Chemists Laboratory Sdn. Bhd. (Malaysia) within 3 days of sampling, in accordance with American Public Health Association and United States Environmental Protection Agency guidelines.

Marine agar (Conda, Torrejón de Ardoz Madrid, Spain) was adjusted to pH 7.6 with 3 M NaOH. A 100 μL aliquot of a water sample was spread on the agar and incubated at 50°C. After 2 days of incubation, several colonies appeared on the agar. Three distinct colonies were repeatedly streaked on the same medium to obtain pure colonies as confirmed by a single morphology and size when examined directly using a DM300 light microscope (Leica Microsystems, Germany). 16S rRNA gene sequences of these isolates were amplified using the forward primer 27F (5′-AGAGTTTGATCMTGGCTCAG-3′) and the reverse primer 1525R (5′-AAGGAGGTGWTCCARCC-3′) (Lane, 1991; Chai et al., 2012). Sequencing was performed at Malaysian First BASE service provider.

The isolated strains were Gram stained and then examined under a Leica DM300 light microscope. For investigation of endospore formation, endospore staining was conducted on bacterial colony obtained from a fresh culture plate as well as a week-old culture (Schaeffer and Fulton, 1933). The growth temperature range of strain RA was assessed by growing this bacterium in marine broth and were incubated at 4, 10, 37, 45, 50, 55, 60, 65, 70, and 75°C for up to 3 days. To determine the optimal pH which support the growth of this bacterium, strain RA was grown in non-buffered marine broths with various pH-values (pH 3.0–12 with interval pH-value of 0.5). The salt tolerance of strain RA was determined in marine broth [2% (w/v) NaCl] and half-strength marine broth [1% (w/v) NaCl], at increments of 1.0% (w/v) up to 20% w/v.

Carbohydrate utilization for strain RA was measured using API 50CHB/E test strips (bioMe'rieux, Marcy-l'Étoile, France). Catalase activity and oxidase activity were determined as described by Cowan and Steel (1965). Motility of strain RA was determined using semi-solid medium and bacterial cells were inoculated with a straight wire making a single stab down the center of the tube to about half the depth of the medium, followed by incubation at 50°C and were examined for up to 3 days. Susceptibility to different antibiotics was determined by the disc diffusion method (Kirby–Bauer antibiotic testing) on marine agar at 50°C. Bacterial lawns were prepared by spreading the cells on plates with sterile cotton swabs dipped in colony suspensions adjusted to a 2.0 McFarland standard. The following antibiotics were tested: ampicillin, bacitracin, chloramphenicol, erythromycin, gentamicin, kanamycin, nalidixic acid, ciprofloxacin, colistin, penicillin, rifampicin, sulfamethoxazole, and vancomycin.

### DNA purification

Cells were scraped from marine agar plates and subjected to genomic DNA extraction using a Qiagen DNeasy Blood and Tissue Kit (Qiagen, Venlo, Netherlands), according to the manufacturer's instructions. A NanoDrop 1000 spectrophotometer (Thermo Scientific, Wilmington, DE, USA) was used to determine the purity (A_260_/A_280_ ratio) and concentration of the DNA, which were 1.98 and 1400 ng μL^−1^, respectively.

### Genome sequencing and annotation

Whole-genome sequencing libraries were prepared using a Nextera DNA Sample Preparation Kit according to the manufacturer's guidelines (Illumina, Inc., San Diego, CA, USA). Paired-end (2 × 100 bp) sequencing was performed on the HiSeq 2500 platform using the HiSeq Rapid SBS Kit v2 (Illumina, Inc., San Diego, CA, USA). Adapter sequence removal, quality trimming, and de novo genome assembly were conducted using CLC Genomics Workbench version 7.5 (CLC Bio, Aarhus, Denmark). The resulting sequences were then annotated with NCBI Prokaryotic Genome Annotation Pipeline version 2.10, using the “best-placed reference protein set” method, and GeneMarkS+ version 3.1, which integrates information regarding protein alignments, frameshifted genes, non-coding RNA sequences, and DNA-specific statistical patterns typical of protein-coding and non-coding regions into gene predictions (Besemer and Borodovsky, 2005). Annotation and KEGG pathway prediction for strain RA were performed using the online service Pathosystems Resource Integration Center (PATRIC; Mao et al., 2015) as well as Integrated Microbial Genomes/Expert Review (IMG/ER; Markowitz et al., 2009). VirulenceFinder version 1.5 (threshold 85%, minimum length 60%; Joensen et al., 2014) and ResFinder version 2.1 (threshold 60%, minimum length 60%; Zankari et al., 2012) were used to predict the presence of putative virulence factors and antimicrobial resistance genes, respectively.

The following genomes were downloaded from GenBank: *Rhodothermus marinus* DSM 4252^T^ (CP001807.1), *S. ruber* DSM 13855^T^ (CP000159.1), and *S. longa* DSM 21114^T^ (ATTH00000000.1). Genome comparisons were performed using the average nucleotide identity (ANI) function of IMG/ER. The complete 16S rRNA gene sequence of strain RA was submitted to GenBank under accession number KU517707.

### Phylogenetic analysis

The 16S rRNA gene sequences phylogenetic tree was constructed using Neighbor-joining method with 1000 bootstrap replicates. All the methods above were conducted using Molecular Evolutionary Genetics Analysis software (MEGA, Version 6.0; Tamura et al., 2013).

### Data access

Sequencing data for strain RA are available online as BioProject PRJNA308615, NCBI taxonomy ID 1779382, Patric genome id 1100069.3, and IMG/ER Taxon ID 2648501586 (GOLD ID Gs0118059). This Whole Genome Shotgun project has been deposited at DDBJ/EMBL/GenBank under the accession number LRDG00000000. The version described in this paper is version LRDG01000000. A Fasta file of the strain RA genome is available in the following figshare link (https://figshare.com/s/60c1a70599d11684aaf7). All Supplementary Materials are available in figshare.

## Results and discussion

### Site description

The hot spring sampled in this study is located near Ayer Hangat (AH) on Langkawi Island, Malaysia. Owing to its relatively low temperature (~45°C), the hot spring has been used for balneotherapy for tourists and local residents. The water in this hot spring is trapped within a man-made pit and is therefore nearly stagnant and is slightly yellowish, and there is a thin biomat (<1 cm) on the sides of the pit that is light brown, green, and yellow. Water samples taken from this site contained high concentrations of Na^+^ (7900 mg L^−1^), Cl^−^ (13,800 mg L^−1^), Mg^2+^ (390 mg L^−1^), SO${}_{4}^{2-}$ (950 mg L^−1^), S (480 mg L^−1^), and CaCO_3_ (5020 mg L^−1^).

### Cell morphology and biochemical analyses

Three bacteria (designated as strains RA, RB, and RC) were isolated from a water sample taken at the AH hot spring. Based on 16S rRNA gene sequences, strains RB and RC had the closest match with *Bacillus* spp. and *Geobacillus* spp., respectively. Since strain RA had low sequence similarity to known *Rhodothermaceae* (see below), it was selected for subsequent analyses. The strain RA colonies appeared smooth, mucoid, and raised, with ~1 mm margins. Furthermore, while the majority of *Rhodothermaceae* strains form orange-reddish colonies (Park et al., 2014), the colonies of strain RA were cream colored (Table 1). Meanwhile, light microscopy analysis demonstrated that this isolate is a gram-negative, rod-shaped bacterium that can be arranged in pairs and chains or as lone cells. *Rhodothermus* spp. are known to occur singly, but not in chains or filaments (Park et al., 2014). Strain RA was motile and catalase positive and it grew well in marine broth and half-strength marine broth but failed to grow on Luria–Bertani, Reasoner's 2A, Mueller–Hinton, or nutrient agar, with or without supplementation with 2% (w/v) NaCl. While the microorganism grew at 37–60°C and pH 5.0–9.0, optimal growth occurred at 50–60°C and under circumneutral conditions. Strain RA was unable to grow at 65°C. Furthermore, strain RA exhibited growth in marine broth supplemented with 1–10% (w/v) NaCl. On marine agar, strain RA was susceptible to erythromycin (zone of inhibition of 25 mm around a 5 μg disc), rifampicin (25 mm zone around a 5 μg disc), and penicillin (35 mm zone around a 10 U disc). Lastly, like *R. marinus* DSM 4252^T^, strain RA is a non-spore-forming bacterium.

**Table 1:** **General features and information regarding the sequencing of the strain RA genome, according to the MIGS (Minimum information about a genome sequence) recommendations**.

**Items**	**Description**
**GENERAL FEATURES**	
Gram stain	Negative
Cell shape	Rod
Pigmentation	Cream
Motility	Motile
Temperature range; optimum	37−60°C; 50−60°C
Salinity range; optimum	1−10.0% NaCl (w/v); 1−5% NaCl (w/v)
pH range; optimum	5.0−9.0; 7.6
Carbon source	Varied
**MIGS 4.0 DATA**	
Submitted_to_insdc	GenBank
investigation_type	bacteria_archaea
project_name	*Rhodothermaceae* bacterium RA genome sequencing
experimental_factor	NA
lat_lon	NA
Depth	NA
geo_loc_name	Langkawi, Malaysia
collection_date	2014-10-10
env_biome	Hot spring
env_feature	Water
env_material	Water with high NaCl content
env_package	Missing
num_replicons	NA
ref_biomaterial	NA
source_mat_id	NA
Pathogenicity	NA
biotic_relationship	Free-living
trophic_level	Chemoheterotroph
rel_to_oxygen	Aerobic
isol_growth_condt	Marine agar, pH 7.6, 50°C
**GENOME ASSEMBLY DATA**	
assembly_name	CLC bio CLC Genomics Workbench version 7.5
finishing_strategy	*De novo* assembly to high quality draft; 121-fold coverage
sequencing Technology	HiSeq 2500

### Phylogenetic relationships

**Figure 1:**
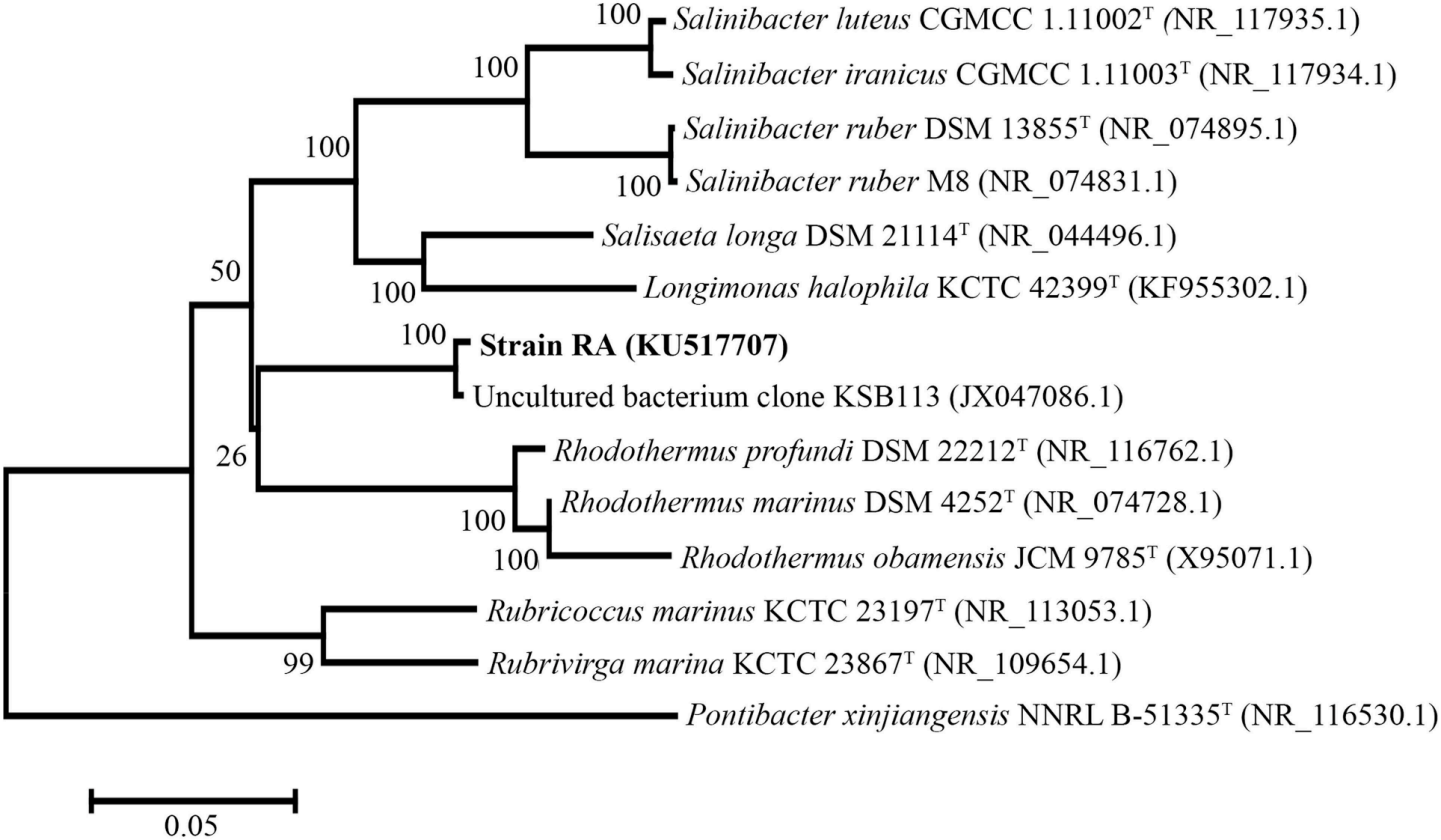
**Phylogenetic tree of strain RA and other *Rhodothermaceae* genera based on 16S rRNA sequencing**. *Pontibacter xinjiangensis* NNRL B-51335^T^ was selected as the out group (https://figshare.com/s/4f470ab9ce3727bf4392).

For phylogenetic analysis of strain RA, we sequenced the entire 16S rRNA coding region (1516 bp) of the microorganism and compared the resulting sequence against the GenBank and EzTaxon-e databases (Kim et al., 2012). These analyses indicated that the closest relative (99% identity, 98% coverage) of strain RA is the uncultured bacterial metagenome clone KSB113 (JX047086), which was identified in a marine hot spring at Kalianda Island, Indonesia (unpublished report). Moreover, the genera closest to strain RA (86.5–89.3%) are those of the family *Rhodothermaceae*: *Rhodothermus* (Alfredsson et al., 1988; Sako et al., 1996; Marteinsson et al., 2010), *Salinibacter* (Antón et al., 2002; Makhdoumi-Kakhki et al., 2012), *Salisaeta* (Vaisman and Oren, 2009), *Rubricoccus* (Park et al., 2011), *Rubrivirga* (Park et al., 2013), and *Longimonas* (Xia et al., 2015; Table 2, Figure 1). The sequence identities of housekeeping genes of strain RA and species of the family *Rhodothermaceae* are also low, for instance (i) *recA*, 83% to *R. marinus* DSM 4252^T^; (ii) *rpoD*, 85% to *R. marinus* DSM 4252^T^ and 82% to *S. ruber* DSM 13855^T^; and (iii) *gyrB*, 79% to *R. marinus* DSM 4252^T^ and 79% to *S. ruber* DSM 13855^T^.

**Table 2:** **General data and comparison of strain RA and other *Rhodothermaceae* genera**.

	**Strain RA**	***Rhodothermus marinus* DSM 4252^T^**	***Salinibacter ruber* DSM 13855^T^**	***Salisaeta longa* DSM 21114^T^**	***Rubricoccus marinus* KCTC 23197^T^**	***Rubrivirga marina* KCTC 23867^T^**	***Longimonas halophila* KCTC 42399^T^**
Taxonomy ID	1,779,382	518,766	309,807	1,089,550	716,817	1,196,024	n.a
Colony color	Cream	Red	Red	Red	Red	Pale-red	Red
Cell shape	Rod	Rod	Rod	Rod	Cocci	Rod	Rod
NaCl range (%; w/v)	1−10	2−6	11−30	5−20	1−5	1−5	4−25
Temperature range	37−60	5−77	37−47	37−46	5−37	10−37	20−50
pH range	5−9	7.0	6.5−8.0	6.5−8.5	5.0−9.0	6.0−9.0	6.5−8.5
DNA GC%	68.3 (genome)	64.3 (genome)	66.5 (HPLC); 66.2 (genome)	62.9 (HPLC); 63.5 (genome)	68.9 (HPLC)	64.8−65.8 (HPLC)	61.5 (HPLC)
16S rRNA similarity to strain RA (%)	100	89.35	86.50	88.83	88.6	88.78	88.14
Genome size (bp)	4,616,094	3,386,737	3,587,328	3,192,765	–	–	–
Predicted coding DNA sequences	3680	2914	3086	2833	–	–	–
Genome contigs no.	91	1	1	3	–	–	–
ANI against strain RA (%)	100	73.28	71.85	71.47	–	–	–
16S rRNA accession number	KU517707	NR_074728	NR_074895.1	NR_044496.1	NR_113053	NR_109654	KF955302.1
Genome DDBJ/EMBL/GenBank accession number	LRDG00000000	CP001807.1	CP000159.1	ATTH00000000.1	–	–	–
GOLD ID	Gp0127843	Gp0001173	Gp0000409	Gp0015133	–	–	–
PATRIC ID	1100069	518766	309807	1089550	–	–	–
KEGG complete genome T number	–	T01119	T00312	–	–	–	–
References	This study	Alfredsson et al., 1988; Nolan et al., 2009	Antón et al., 2002; Mongodin et al., 2005	Vaisman and Oren, 2009	Park et al., 2011	Park et al., 2013	Xia et al., 2015

We then compared the available genome sequences of several of these strains with the draft genome of strain RA (Table 2). In addition to exhibiting low 16S rRNA gene sequence identity (89.3%), strain RA and *R. marinus* DSM 4252^T^ had a low average ANI-value of 73.28 (Table 2). The ANI-value between strain RA and *R. marinus* SG0.5JP17-171 or *R. marinus* SG0.5JP17-172 was 73.23, while that between strain RA and *R. profundi* DSM 22212^T^ was 70.66. Meanwhile, the average ANI-values for strain RA when compared with *S. ruber* DSM 13855^T^ and *S. longa* DSM 21114^T^ were 71.85 and 71.47, respectively. Collectively, these data strongly suggest that strain RA is a novel strain of *Rhodothermaceae*.

### Genomic features of strain RA

**Figure 2:**
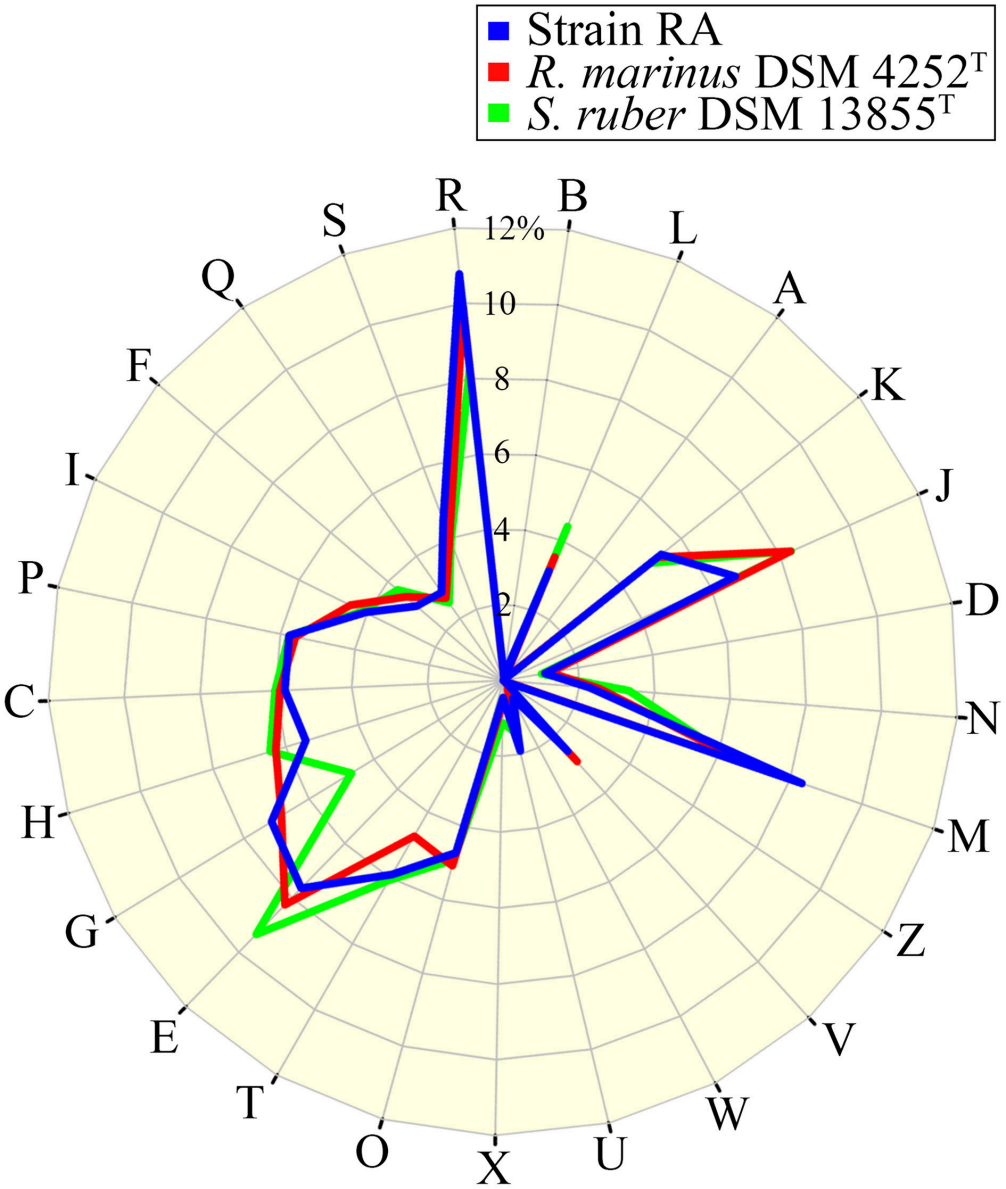
**Percentage of CDS involved in COG classifications for strain RA, *R. marinus* DSM 4252^T^ and *S. ruber* DSM 13855^T^**. B, Chromatin structure and dynamics; L, replication, recombination, and repair; A, RNA processing and modification; K, transcription; J, translation, ribosomal structure, and biogenesis; D, cell cycle control, cell division, chromosome partitioning; N, cell motility; M, cell wall/membrane/envelope biogenesis; Z, cytoskeleton; V, defense mechanisms; W, extracellular structures; U, intracellular trafficking, secretion, and vesicular transport; X, mobilome, prophages, transposons; O, post-translational modification, protein turnover, chaperones; T, Signal transduction mechanisms; E, amino acid transport and metabolism; G, carbohydrate transport and metabolism; H, coenzyme transport and metabolism; C, energy production and conversion; P, inorganic ion transport and metabolism; I, lipid transport and metabolism; F, nucleotide transport and metabolism; Q, secondary metabolites biosynthesis, transport and catabolism; S, function unknown; and R, general function prediction only (https://figshare.com/s/8a2ccd9f2b7aa439f040).

The draft genome of strain RA is 4,616,094 bp in length (Table 3), and the largest contig is 819,789 bp. The N75, N50, and N25-values are 89,274, 152,113, and 242,570 bp, respectively. The average coverage for the 91 contigs obtained was 120-fold. Notably, the genome of strain RA is larger than those of *Rhodothermus marinus* (3.3 Mbp), *S. ruber* (3.5–3.8 Mbp), and *S. longa* (3.1 Mbp; a draft genome with three contigs; Table 2). Furthermore, the genome is predicted to contain 3680 coding DNA sequences (CDS) and 50 rRNA genes, and it has an average GC content of 68.28%, which is markedly higher than that of *R. marinus* DSM 4252^T^ (64.3%; Nolan et al., 2009), *S. ruber* DSM 13855^T^ (66.2%; Mongodin et al., 2005), and *S. longa* DSM 21114^T^ (63.5%) but comparable to that of *R. marinus* KCTC 23197^T^ (68.9%; Table 2). Among the CDS, the functions of 2815 (76.49%) sequences could be predicted, while 879 (24.38%) of the total CDS are annotated as enzymes. Protein-coding genes related to clusters of orthologous groups (COGs) are shown in Figure 2.

**Table 3:** **Genome statistics of strain RA**.

	**Number**	**% of Total (%)**
DNA, total number of bases	4,616,094	100.00
DNA coding number of bases	4,160,472	90.13
DNA G+C number of bases	3,152,009	68.28
DNA scaffolds	61	100.00
CRISPR count	5	
Genes total number	3680	100.00
Protein coding genes	3630	98.64
RNA genes	50	1.36
rRNA genes	3	0.08
5S rRNA	1	0.03
16S rRNA	1	0.03
23S rRNA	1	0.03
tRNA genes	44	1.20
Other RNA genes	3	0.08
Protein coding genes with function prediction	2815	76.49
Without function prediction	815	22.15
Protein coding genes with enzymes	897	24.38
w/o enzymes but with candidate KO based enzymes	50	1.36
Protein coding genes connected to Transporter Classification	365	9.92
Protein coding genes connected to KEGG pathways3	912	24.78
Not connected to KEGG pathways	2718	73.86
Protein coding genes connected to KEGG Orthology (KO)	1587	43.12
Not connected to KEGG Orthology (KO)	2043	55.52
Protein coding genes connected to MetaCyc pathways	777	21.11
Not connected to MetaCyc pathways	2853	77.53
Protein coding genes with COGs	2222	60.38
With KOGs	713	19.38
With Pfam	2905	78.94
With TIGRfam	1144	31.09
With InterPro	1900	51.63
With IMG Terms	611	16.60
With IMG Pathways	231	6.28
With IMG Parts List	246	6.68
In internal clusters	734	19.95
In chromosomal cassette	3633	98.72
Chromosomal cassettes	309	-
Biosynthetic clusters	14	-
Genes in biosynthetic clusters	236	6.41
Fused protein coding genes	93	2.53
Protein coding genes coding signal peptides	527	14.32
Protein coding genes coding transmembrane proteins	881	23.94
COG clusters	1399	62.96
KOG clusters	485	21.83
Pfam clusters	1958	67.40
TIGRfam clusters	916	80.07

Using the Phylogenetic Distribution of Genes function in IMG/ER, we determined the best hits for protein-coding genes in strain RA at a 60% identity cutoff. A total of 1408 genes (39% of the protein-coding genes) were found to be similar to genes in the phylum *Bacteroidetes*. The majority of these sequences are affiliated with class *Cytophagia* and unclassified classes, while only 52 genes are related to *Bacteroidia, Flavobacteriia*, and *Sphingobacteriia*. Table S1 shows that 1987 genes (54.7% of the protein-coding genes) were not assigned to any phylum, as they are specific to strain RA. When all protein-coding genes were examined at a 90% sequence identity threshold, 99.75% (3621) of the genes were classified as unassigned. This suggests that the gene sequences of strain RA are more diverse than those of its *Rhodothermaceae* counterparts (data not shown).

Consistent with its observed motility, strain RA carries the genes necessary for complete flagellum assembly. Furthermore, genomic analysis using the antiSMASH predictor program (Blin et al., 2013) identified a gene cluster encoding a type III polyketide synthase. The key enzyme within this cluster has a structural domain related to chalcone synthase; thus, strain RA might use this enzyme to produce certain types of aromatic ketones, including chalcones, which possess important properties for industrial use (Singh et al., 2014).

### Comparison of the genome of strain RA with those of other rhodothermaceae strains

At the time of writing, the genomes of three *R. marinus* strains and two *S. ruber* strains, as well as the draft genome of *S. longa* DSM 21114^T^, were publicly available. Unless otherwise specified, we chose to compare the genome of strain RA with the complete genome sequences of *R. marinus* DSM 4252^T^ and *S. ruber* DSM 13855^T^.

RAST analysis identified 215 and 221 strain RA genes that are not carried by *R. marinus* DSM 4252^T^ and *S. ruber* DSM 13855^T^, respectively (Table S2), including genes encoding DnaK-related proteins, cytochrome C oxidase subunit CcoN, superfamily II DNA/RNA helicases (SNF2 family), primosomal protein N′ (replication factor Y) superfamily II helicase, β-ureidopropionase, fucose permease, gluconolactonase, α-l-fucosidase, malto-oligosyltrehalose synthase, α-1,2-mannosidase, endo-1,4-β-xyla nase, endoglucanase, choline-sulfatase, putrescine transport ATP-binding protein PotA, arylsulfatase, and sulfate permease. Of these, strain RA likely maintains the genes for sulfate permease and arylsulfatase to withstand the high sulfur and sulfate contents of the AH hot spring. Furthermore, the presence of these genes suggests that strain RA plays a role in the recycling of sulfur within the hot spring. Two cholesterol oxidase genes were also present in strain RA but not in closely related genera. Notably, cholesterol oxidases catalyze the conversion of cholesterol to cholest-4-en-3-one, which has been proposed as a potential drug candidate for treatment of amyotrophic lateral sclerosis (Bordet et al., 2007).

KEGG metabolic pathway analyses indicated that the starch and sucrose metabolism pathways of strain RA are similar to those of *Rhodothermus* and *Salinibacter* spp. According to these analyses, strain RA is capable of hydrolyzing starch to maltose, glucose, or trehalose and converting starch to glycogen. Meanwhile, all three genera encode the enzymes (EC 3.2.1.4 and 3.2.1.21) necessary for hydrolysis of cellulose to glucose Figure S1.

Likewise, *R. marinus* DSM 4252^T^, *S. ruber* DSM 13855^T^, and strain RA each encode a type I system (TolC), a Sec-SRP pathway (SecD/F, SecY, SecA, YidC, FstY, Ffh), and a Tat pathway (TatC) for protein secretion. However, while strain RA also carries genes encoding type II (GspD, GspE, GspF) and type VI (VgrG, Hcp, IcmF, DotU, ClpV) secretion systems, virulence factor prediction analyses using the PATRIC database (Wattam et al., 2014), VirulenceFinder 1.5 (Joensen et al., 2014), and ResFinder 2.1 (Zankari et al., 2012) indicated that this strain is likely non-pathogenic.

Certain ABC transporters encoded by strain RA, including those specific for phosphate (PstS, PstA, PstB, PstC), lipoproteins (LolC, LolE, LolD), and iron-siderophores (FhuB, FhuC, FhuD), are potentially produced by *R. marinus* DSM 4252^T^ and *S. ruber* DSM 13855^T^. In addition, there were multiple transporters that were encoded in the genomes of strain RA and *R. marinus* DSM 4252^T^ but not in that of *S. ruber* DSM 13855^T^, including transporters for molybdate (ModA, ModB), iron(III; AfuA, AfuB), zinc/manganese/iron (TroA, TroB, TroC, TroD), raffinose/stachyose/melibiose (MsmK), sorbitol/mannitol (SmoK), cellobiose (MsiK), chitobiose (MsiK), arabinooligosaccharide (MsmX), and glucose/mannose (MalK; Table S2). Meanwhile, strain RA also encodes an RbsABC transporter for uptake of ribose/xylose. Lastly, strain RA contains genes encoding unique CusS, CusR, CusB, and CusA proteins that are predicted to play a role in copper and/or silver efflux, which are absent from all currently known *Rhodothermus* and *Salinibacter* spp.

### Temperature adaptation genes of strain RA

The average water temperature of the AH hot spring during sampling was 45°C; however, *in vitro* analysis demonstrated that strain RA is capable of thriving at temperatures up to 60°C. Meanwhile, analysis of the composition of the spring water detected high levels of NaCl. We therefore endeavored to elucidate the mechanisms by which strain RA withstands thermal and osmotic stresses by characterizing the stress-related genes within the annotated genome.

The linear polyamines putrescine and spermidine have been associated with the thermophilicity of thermophiles (Takahashi and Kakehi, 2010; Goh et al., 2014). Notably, in addition to harboring the genes necessary to synthesize putrescine via *N*-carbamoylputrescine amidase, strain RA appears to be capable of taking up putrescine and spermidine from the environment using the PotA and PotC transporters. In contrast, the genes encoding PotA and PotC are not present in the genomes of *R. marinus* DSM 4252^T^ and *S. ruber* DSM 13855^T^. A unique sequence in strain RA was annotated as a primosomal protein N′ (replication factor Y) superfamily II helicase gene, and the corresponding protein sequence exhibited >58% sequence identity to PriA helicases deposited in GenBank. Several of these helicases have been proposed to play important roles in the thermostability of certain thermophiles (Goh et al., 2014). As mentioned earlier, the GC content of the strain RA genome is higher than that of most other genomes of *Rhodothermaceae*. In some cases, high genome and tRNA GC% levels are associated with increased optimal growth temperatures (Trivedi et al., 2005). Yet, the average tRNA GC content (61.4%) of strain RA is slightly lower than that of the rest of the genomes (68.3%). Additionally, the strain RA genome contains CDS that exhibit sequence similarity to genes encoding chaperones (DnaJ, DnaK, HtrA, metal chaperone) and the known heat shock proteins GroES and GroEL (HSP-60 family co-chaperones), and it contains three copies of the gene encoding the cold shock protein CspA. These CspA proteins exhibited the highest level of sequence identity to counterparts from *R. marinus* DSM 4252^T^ (67, 78, and 80%, respectively).

### Osmotic stress adaptation genes of strain RA

We detected several genes encoding proteins involved in adaptation to osmotic stress within the strain RA genome, including Na^+^/H^+^ antiporters, a sodium/solute symporter, and a sodium/glutamate symporter (Table S3). Moreover, we detected three separate trehalose synthase genes (EC 5.4.99.16), which exhibited 64–65% identity to genes present in *S. longa* DSM 21114^T^ and *S. ruber* DSM 13855^T^, while similar genes were absent from the *R. marinus* DSM 4252^T^ genome. Strain RA also harbors a gene predicted to encode malto-oligosyltrehalose trehalohydrolase (EC 5.3.2.141) that is 58% similar to a gene present in *S. ruber* DSM 13855^T^. It also has a malto-oligosyltrehalose synthase (EC 5.4.99.15) gene that exhibits 58% sequence identity to a CDS present in *Truepera radiovictrix* but has no homologs in the genomes of *S. longa* DSM 21114^T^, *S. ruber* DSM 13855^T^, and *R. marinus* DSM 4252^T^. In addition, strain RA is predicted to be capable of biosynthesizing the osmoprotectant choline via a choline-sulfatase (EC 3.1.6.6). However, unlike *S. ruber* DSM 13855^T^, both strain RA and *R. marinus* DSM 4252^T^ lack the genes encoding the osmoprotectant-associated transporters ProX, ProW, ProV, OpuBC, OpuBB, and OpuBA. Lastly, strain RA encodes the potassium uptake proteins TrkA and TrkH, the osmotically inducible protein C, and the K^+^ channel histidine kinase KdpD but lacks genes encoding a glycine betaine transporter or proteins involved in ectoine transport/synthesis.

## Conclusions

In this report, we have described the genome of strain RA, which was isolated from a saline hot spring in Malaysia. The draft genome of strain RA comprises 4,616,094 bp with a mean GC content of 68.3%. It contains 91 contigs with an N50 contig length of 152,113 bp. This genome is predicted to include 3630 protein-coding genes. At a 60% identity cutoff, a low percentage of these protein-coding genes are similar to genes in the phylum *Bacteroidetes*. The results of 16S rRNA gene sequencing, ANI-value and genome comparisons clearly indicate that this strain exhibits many differences from known genera of *Rhodothermaceae*. In the NCBI taxonomy database, the lineage for strain RA is classified as Bacteria; FCB group; Bacteroidetes/Chlorobi group; Bacteroidetes; Bacteroidetes Order II. Incertae sedis; *Rhodothermaceae*; unclassified *Rhodothermaceae*. In the near future, chemotaxonomic and phenotypic characterization of strain RA will be performed to further compare this strain with other related type strains from the family *Rhodothermaceae*, and names for the genus and species will be proposed.

## Author contributions

KG and KC designed the experiments and wrote manuscript; SL, KL, CC, RE, MS, and TA performed biochemical and sequencing experiments, and analyzed the genetic content of the bacterium.

### Conflict of interest statement

The authors declare that the research was conducted in the absence of any commercial or financial relationships that could be construed as a potential conflict of interest.
