# Supplementary material for: Genome Analysis of a New Rhodothermaceae Strain Isolated from a Hot Spring
Source: Front Microbiol. 2016 Jul 14;7:1109. doi: 10.3389/fmicb.2016.01109 (PMC4943939; doi:10.3389/fmicb.2016.01109)
Supplement: Supplementary file 3 [file DataSheet3.pdf]

Supplementary Material

Genome Analysis of a New *Rhodothermaceae* Strain Isolated from a Hot Spring

Kian Mau Goh[1,*], Kok-Gan Chan[2], Soon Wee Lim[1], Kok Jun Liew[1], Chia Sing Chan[1], Mohd Shahir Shamsir[1], Robson Ee[2], Tan-Guan-Sheng Adrian[2]

*** Correspondence:** Kian Mau Goh: gohkianmau@utm.my

TABLE S1 | Phylogenetic distribution of best hit of strain RA proteins to other phylum at 60% identity cutoff.

Domain	Phylum	No. of sequence	Percentage, %
Archaea	*Euryarchaeota*	1	0.03
Bacteria	*Acidobacteria*	9	0.25
Bacteria	*Actinobacteria*	6	0.17
Bacteria	*Armatimonadetes*	4	0.11
Bacteria	*Atribacteria*	1	0.03
Bacteria	BRC1	1	0.03
Bacteria	*Bacteroidetes*	1,408	38.79
Bacteria	*Chlorobi*	1	0.03
Bacteria	*Chloroflexi*	23	0.63
Bacteria	*Cloacimonetes*	1	0.03
Bacteria	*Cyanobacteria*	19	0.52
Bacteria	*Deinococcus-Thermus*	22	0.61
Bacteria	*Firmicutes*	10	0.28
Bacteria	*Gemmatimonadetes*	16	0.44
Bacteria	*Hydrogenedentes*	1	0.03
Bacteria	*Ignavibacteriae*	5	0.14
Bacteria	*Latescibacteria*	4	0.11
Bacteria	*Marinimicrobia*	1	0.03
Bacteria	*Planctomycetes*	16	0.44
Bacteria	*Proteobacteria*	75	2.07
Bacteria	*Synergistetes*	1	0.03
Bacteria	*Verrucomicrobia*	8	0.22
Eukaryota	Unclassified	1	0.03
-	Unassigned	1,987	54.74

TABLE S2 | List of unique strain RA genes absent from the genomes of *Rhodothermus marinus* DSM 4252^{T} and *Salinibacter ruber* DSM 13855^{T}. (https://figshare.com/s/0272e915c6e76c015723)

TABLE S3 | Strain RA proteins associated with osmotic or general stress adaptation. (https://figshare.com/s/708eda923332c22d863f)

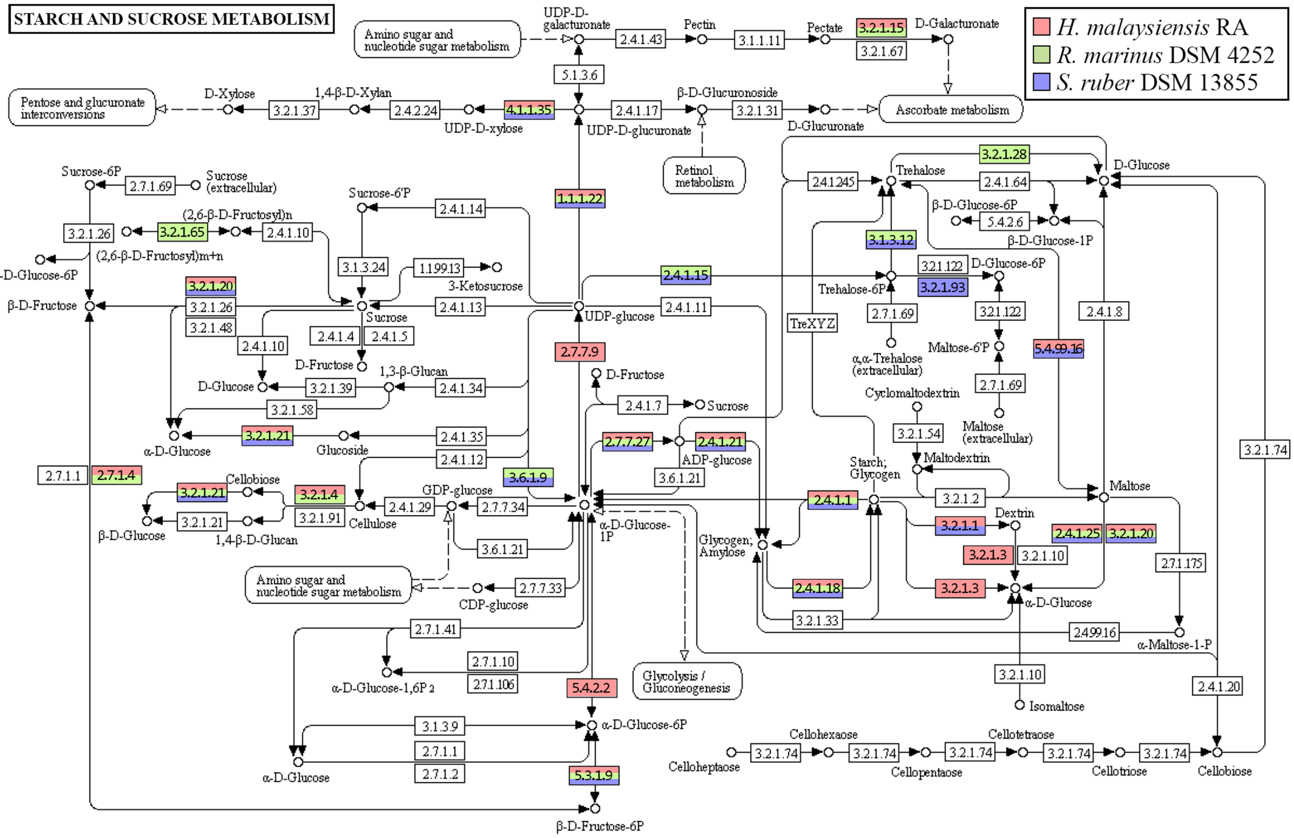

FIGURE S1 | KEGG-based representation of starch and sucrose metabolism in strain RA, *Rhodothermus marinus* DSM 4252^{T}, and *Salinibacter ruber* DSM 13855^{T}. (https://figshare.com/s/6af28898b3131a0e5c5e)
